# Dietary Carnosine Supplementation in Healthy Human Volunteers: A Safety, Tolerability, Plasma and Brain Concentration Study

**DOI:** 10.3390/nu17132130

**Published:** 2025-06-27

**Authors:** Ali N. Ali, Li Su, Jillian Newton, Amy K. Grayson, David Taggart, Simon M. Bell, Sheharyar Baig, Iain Gardner, Barbora de Courten, Arshad Majid

**Affiliations:** 1 Sheffield Institute for Translational Neuroscience, University of Sheffield, Sheffield S10 2TN, UK; l.su@sheffield.ac.uk (L.S.); sbaig1@sheffield.ac.uk (S.B.); 2 Combined Community and Acute Care Group, Sheffield Teaching Hospitals NHS Foundation Trust, Sheffield S10 2JF, UK; 3 Department of Psychiatry, University of Cambridge, Cambridge CB2 1TN, UK; 4 Biomolecular Science Research Centre, Sheffield Hallam University, Sheffield S1 1WB, UK; jillian.newton@shu.ac.uk (J.N.);; 5 Glycyx MOR, Inc., San Francisco, CA 94121, USA; david.taggart@glycyx.com; 6 Translational DMPK Science, Certara UK Limited, Sheffield S1 2BJ, UK; iain.gardner@certara.com; 7 School of Health and Biomedical Sciences, Royal Melbourne Institute of Technology University, Melbourne 3000, Australia; barbora.decourten@monash.edu

**Keywords:** carnosine, histidine, safety and tolerability, pharmacokinetics, brain concentration

## Abstract

Background: Carnosine is a multimodal pleotropic endogenous molecule that exhibits properties that make it a compelling therapeutic agent for further evaluation in a number of diseases. However, little data currently exists on its pharmacokinetic profile, maximum tolerated doses, side effects and whether oral administration can lead to elevated brain concentrations. Method: To investigate this, sixteen healthy volunteers underwent a single dose-escalation study of oral carnosine to establish safety, tolerability, and pharmacokinetics. A subset (*n* = 5) underwent Proton Magnetic Resonance Imaging (MRI) spectroscopy to evaluate the effect of oral dosing on brain carnosine concentrations, and another subset (*n* = 4) completed a long-term (4-week) dosing study. Results: Oral carnosine was safe and well tolerated up to a dose of 10 g. At doses of 15 g, the frequency of adverse events became unacceptably high, with 77% of participants experiencing side effects, most commonly headache (43.5%), nausea (21.7%) and paraesthesia (21.7%). While pharmacokinetic profiles varied between individuals, peak plasma concentrations occurred within the first hour of dosing. Little circulating carnosine was detectable beyond 4 h. Brain carnosine concentration increased at 1 h post-dose but reverted to baseline values by 5 h. Long-term dosing at 5 g twice daily did not result in any adverse events. Conclusions: Our data will inform dosing interventions in future clinical trials of this exciting agent.

## 1. Introduction

Carnosine is an endogenous dipeptide, composed of two amino acids, beta-alanine and L-histidine (β-alanyl-L-histidine), that was first discovered in 1900 as a constituent of meat [1]. It is found in high abundance in skeletal and cardiac muscle and in lower quantities in other tissues such as the brain [1,2]. Carnosine (CAR) has been used extensively as a dietary supplement by athletes to delay muscle fatigue and boost performance [3]. However, CAR has several other properties which play important roles in normal physiological functions including PH buffering [2], endogenous free radical scavenging [4], anti-glycating functions [5] and anti-inflammatory [6] and metal ion-chelating effects [7]. These properties also make CAR a promising agent in the field of neuroprotection and neurodegeneration. Indeed, in vitro and in vivo preclinical models have demonstrated the favourable effects of CAR in limiting neuronal injury in acute stroke [8,9] and preventing aggregation of amyloid-beta and alpha-synuclein proteins in Alzheimer’s and Parkinson’s disease models, respectively [10,11]. This has led to considerable interest in investigating CAR in clinical populations of neurodegeneration [12,13]. However, it is unclear whether the doses used in preclinical rodent studies are needed in clinical populations and whether the rodent doses can be achieved in humans without inducing unacceptable side effects.

Much of the CAR in humans is synthesised in vivo from beta-alanine, itself synthesised in the liver or absorbed from the diet, and L-histidine, which must be absorbed [14]. However dietary absorption of CAR can also influence circulating concentrations significantly. CAR is broken down (hydrolysed) into its constituent amino acids by carnosinases that are narrowly specific found in the plasma (CN1), and those more broadly specific found in cytosolic tissue (CN2), at varying concentrations [15]. The tissue distribution of CAR in humans at baseline and how dietary intake of CAR affects tissue distribution are not clearly understood. Using high-performance liquid chromatography (HPLC), Park et al. showed that no plasma CAR was detectable in individuals at baseline but oral administration of beef containing 0.248 g of carnosine resulted in a maximum level of 32.7 mg/L, 2.4 h after administration [16]. Everaert et al. showed that rises in plasma CAR occurred only in individuals with lower plasma carnosinase activity [17]. They recorded peak concentrations as high as 200 μM using the highest published dose of oral CAR supplementation in a human study (60 mg/Kg). However, in both studies, the measured plasma values may have been influenced by continued carnosinase degradation of CAR in withdrawn plasma samples during processing. Studies on whether higher doses of CAR are safe or well tolerated and how quickly they are cleared are sparse. Furthermore, there is even less data available on whether oral CAR supplementation leads to detectable changes in brain CAR levels. This is of significant importance if CAR is to be considered a potential therapeutic agent in neuroprotection or neurodegenerative disease.

In this study of healthy human volunteers, we aimed to establish the safety and tolerability of differing oral doses of CAR and understand how this affects plasma and brain tissue concentrations.

## 2. Materials and Methods

### 2.1. Study Design and Participants

This was a single-centre, open-label dose-escalation study in an anthropometrically diverse sample of healthy volunteers to determine the CAR concentration in the plasma and brain before and after dietary supplementation with CAR. Plasma levels were measured using liquid chromatography mass spectroscopy (LC-MS), and in a sub-group of volunteers, brain levels were measured non-invasively using Proton Magnetic Resonance Imaging (MRI) spectroscopy at the highest tolerated dose. These data provide pharmacokinetics (PK) of CAR in the plasma and brain. The study protocol was approved by the University of Sheffield local research ethics committee and conducted at the Clinical Research Facility (CRF) at the Royal Hallamshire Hospital, Sheffield, UK, between June 2021 and January 2022. Volunteers were recruited from staff and student mailing lists; inclusion and exclusion criteria are detailed in Table 1. Potential participants were reviewed at a screening visit where consent was obtained, a medical history was reviewed by a clinician, and a Short Food Intake assessment completed, alongside the measurement of heart rate (HR), blood pressure (BP), and respiratory rate (RR). A screening blood sample was taken to exclude any serious haematological or biochemical abnormalities (full blood count, urea and electrolytes, liver function tests, calcium, magnesium, phosphate, thyroid-stimulating hormone). A total of 16 healthy volunteers provided written informed consent and were recruited to the study, of which 5 were also recruited to the MRI spectroscopy brain concentration sub-study, and 4 were recruited to a long-term dosing study.

### 2.2. Study Protocol

We aimed to assess the safety, tolerability and PK of 4 g, 6 g, 10 g, 15 g, and 20 g of oral CAR supplementation. Participants attended the CRF for the 4 g dose study first and then returned for the next escalating dose (6 g) after a 2-week ‘washout’ period if they had not experienced any safety or tolerability concerns at the 4 g dose. Similarly, they then returned every 2 weeks for the next dose escalation (10 g, 15 g, 20 g) if no safety or tolerability problems were identified at the prior dose. Five individuals were sequentially invited to participate in the MRI spectroscopy brain concentration study which involved another visit to undertake pre- and post-CAR brain scans at the highest tolerated dose. A further 4 participants were recruited to a long-term dosing study at the highest tolerated daily dose to establish the safety and tolerability of a 4-week course of twice-daily oral carnosine.

At each study visit, participants arrived fasted overnight at the CRF, a catheter was inserted into the antecubital fossa, and the first blood sample was taken for baseline measurements of CAR, beta-alanine (B-ALA), and L-histidine (HIS). Baseline clinical assessments of HR, BP and RR were taken, and the oral dose of CAR was given (time 0, approx 9 am). At the first visit, 4 g of L-carnosine powder (Purebulk Inc., Roseburg, OR, USA) was mixed with 200–400 mL of water and ingested as a single dose. Thereafter, blood samples for CAR, B-ALA and HIS were taken at 15 min, 1 h, 4 h and 8 h after ingestion. HR, BP and RR were recorded every 15 min for the first hour and then every hour until the last blood sample was taken. At 8 h a safety blood sample was taken measuring full blood count and biochemical profiles (urea and electrolytes, liver function tests, serum calcium and phosphate). Participants were observed in the CRF for the duration of the 8 h study day for any side effects. Participants consumed a breakfast that equated to a calorie count of 25–30% (392 ± 45 Kcal) of their resting metabolic rate calculated according to Mifflin [18] approximately 2 hrs after the CAR dose. Participants were advised they could bring their own food in for breakfast (cereals, toast, yoghurt, fruit) and lunch or choose from a selection at the CRF. This aimed to mimic usual breakfast intake, variation of which could potentially influence CAR absorption. Dietary intake was recorded for each individual at breakfast and lunch. Telephone calls to participants 24 h after ingestion of the CAR dose enquired as to the resolution of side effects or the development of late side effects (up to 24 h post-dose).

In the event of no dose-limiting toxicity (DLT, see Section 2.3) observed during a dose, and no other safety concerns, as judged by a data monitoring committee (DMC; made up of 2 independent neuroscience clinicians), the next dose escalation would occur, and the process above was repeated for 6 g, 10 g, 15 g, and 20 g.

For the MRI spectroscopy brain concentration sub-study, 5 participants attended after another 2-week washout period, after an overnight fast, and underwent baseline MRI spectroscopy scanning. Following this, they then ingested the same maximum tolerated single dose of CAR (10 g). They then underwent repeat MRI brain scanning 1 h and 5 h after the baseline and were again monitored in the same way as previously described.

For the long-term dosing sub-study, 4 participants attended after their 2-week washout period to undergo a twice-daily dosing of oral CAR equating to their maximum tolerated dose for 4 weeks (5 g twice daily). They were provided with and taught how to mix and take the oral CAR at home and attended each week for safety blood tests (similar to screening tests), review of compliance, and review of adverse events.

### 2.3. Safety and Tolerability

Any adverse events (AEs) experienced by participants following CAR ingestion, either while on the CRF or once they had returned home within 24 h, were recorded and their severity graded by participants according to a 5-point Likert scale (1 = mild to 5 = very severe). Each AE was reviewed by the PI and causality to CAR assessed. A serious adverse event (SAE) occurs if an AE results in death, is life-threatening, requires hospitalisation, results in persistent disability or incapacity, results in a congenital anomaly or birth defect, or is otherwise considered medically significant by study investigators [19].

Safety of a dose is defined as the absence of any SAE related to CAR, and tolerability is defined as 4 or fewer individuals reporting any AEs of common terminology criteria (CTC) with any severity score. Safety blood tests assessed at 8 h post-dose also identified significant haematological or biochemical abnormalities (falling outside of normal ranges for the local population) that developed following CAR. If safety and tolerability criteria were not met for a specific dose, or blood test abnormalities occurred, this constituted dose-limiting toxicity (DLT) and progression to the next dose escalation would not occur.

### 2.4. Analysis of Plasma Carnosine, Beta-Alanine and L-Histidine

Blood samples taken for CAR analysis are at risk of further degradation by carnosinases during sample processing. This may lead to underestimations of CAR levels and overestimations of levels of B-ALA and HIS [20]. To mitigate these effects, blood samples were drawn from the antecubital fossa into ice-cooled EDTA tubes pre-treated with carnostatine, a carnosinase inhibitor. For each container, 1.5 mg of carnostatine was mixed with 1.5 mL of 0.9% saline (1 mg/mL), which meant that following the addition of 4 mL of whole blood, the final sample concentration of carnostatine was approximately 50 μM. Samples were immediately cooled again using ice (<4 °C) while transported to the laboratory and spun in a refrigerated (4 °C) centrifuge (Eppendorf Centrifuge 5804R) at 13,000 rpm for 10 min to prepare plasma for storing at −80 degrees. The anti-coagulant EDTA also chelates Zn^2+^, which is required for the enzymatic function of carnosinases [21]. We have demonstrated that this method preserves over 90% of the available CAR in the sample compared to approximately 20% without carnostatine (Supplementary Materials). All chemicals and reagents were of analytical grade and purchased from Merck (Dorset, UK).

### 2.5. Preparation of Calibration Standards and Quality Controls

First, 500 μM working solutions of both standards (L-carnosine, L-histidine, B-alanine) and internal standards (L-carnosine-d4, DL-histidine-d3, and β-alanine-3C^13^ N^15^) were prepared in high-performance liquid chromatography (HPLC)-grade water with 0.1% formic acid. Serial dilutions of each were prepared to create a series ranging from 0.05 to 100 µM. Two blank samples of 60% acetonitrile with 0.1% formic acid were analysed at the beginning and end of each experimental run. Blank samples were also injected after each of the three standard curves, and after every 10 samples analysed.

### 2.6. Extraction of Carnosine and Metabolites from Plasma Samples

Frozen plasma samples were thawed at room temperature, vortex-mixed and centrifuged at 13,000 rpm for 10 min at 4 °C. Then, 25 µL of supernatant was removed and 25 µL of each internal standard (500 µM DL-histidine-d3, L-carnosine-d4, β-alanine-3C^13^,N^15^ prepared in HPLC-grade water with 0.1% formic acid) was added, to give a final volume of 100 µL. Samples were vortex-mixed and centrifuged at 13,000 rpm for 10 min at 4 °C. Then, 150 µL of acetonitrile with 0.1% formic acid solution was added to each sample. Samples were then vortex-mixed and centrifuged at 13,000 rpm for 20 min at 4 °C. Finally, 50 µL of supernatant was then mixed with 50 µL of 60% acetonitrile containing 0.1% formic acid and placed in liquid chromatography mass spectrometry vials for analysis.

### 2.7. Liquid Chromatography Mass Spectrometry (LC-MS)

The analysis was carried out using a multiple reaction monitoring (MRM) assay using liquid chromatography (LC)-coupled tandem mass spectrometry with an electrospray ionisation (ESI) source. Chromatographic separation was carried out using an Agilent Infinity II 1290 HPLC system with an XBridge BEH Amide 130 Å, 5 µM, 4.6 × 150 mm column. Mobile phases A and B were 100% HPLC-grade water with 0.1% formic acid and 100% acetonitrile with 0.1% formic acid, respectively. A gradient elution profile was utilised starting with 20% to 5% B for 0.0–0.3 min, held at 5% B for 2 min, 5% B to 90% over 0.7 min then held at 90% B for 10 min. Injection volumes and flow rates were set at 10 uL and 0.4 mL/min, respectively. Analytes were analysed using an Agilent Ultivo triple-quadrupole mass spectrometer with an Agilent Jet Stream Electrospray Ionisation (AJS-ESI) source. Electrospray ionisation was performed in positive polarity mode, with source parameters as follows: sheath gas temperature = 250 °C; sheath gas flow rate = 11 L/min; desolvation gas temperature = 300 °C; desolvation gas flow rate = 7 L/min; nebuliser gas pressure = 15 psi; capillary voltage = 4000 V; nozzle voltage = 1500 V. The mass spectrometer was operated in dynamic multiple reaction monitoring (dMRM) mode. The optimised potentials and transitions used can be found in the Supplementary Materials. Data acquisition and analysis were achieved using MassHunter Acquisition and Qualitative Analysis version B.0.10.0.

### 2.8. Brain Proton MRI Spectroscopy for L-Carnosine

**Figure 1:**
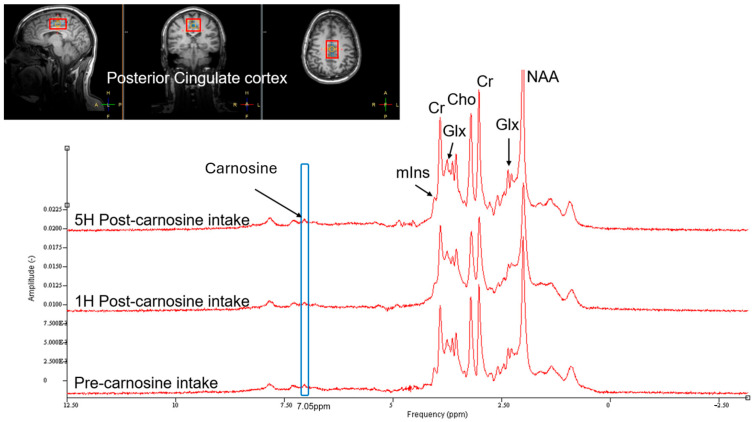
Example of brain MRI spectroscopy spectra for determining carnosine concentration in the posterior cingulate cortex. Spectra for baseline, 1 h and 5 h post-10 g dose carnosine superimposed for comparison. Cr = creatine; Glx = glutamate–glutamine; Cho = choline; NAA = N-acetylaspartate; mlns = myo-inositol.

Following completion of the standard dosing study, and after we had identified the maximum tolerated dose before DLT was observed, 5 participants completed the MRI spectroscopy brain study. On the day of study, participants arrived fasted and underwent a baseline MRI scan of the brain. Following this, they ingested the oral CAR and had repeat MRI brain scans at 1 h and 5 h post-dose. In vivo MRI spectroscopy of the posterior cingulate cortex was acquired at 3 Tesla (3T) using a transmit–receive 8-channel surface coil (Philips Healthcare, Best, The Netherlands). The spectroscopy sequence was a single-voxel stimulated echo acquisition (STEAM) (voxel size 2.5 × 3 × 4 cm^3^) with TE/TR = 10/2000 ms, spectral bandwidth 2000 Hz, 2048 sample points at 160 averages and a central frequency set to 8 ppm [22]. Quantification of CAR was performed using jMRUI 6.0. The time domain signal was multiplied by a −1.5 Hz exponential function prior to Fourier Transformation, and then a 3 Hz Gaussian filter was applied. Subtraction of the residual water signal allowed automatic zero and first-order phasing application. Quantification of the peak at 7.05 ppm was chosen for CAR [23], and peaks were expressed as signal-to-noise ratios (Figure 1).

### 2.9. Pharmacokinetics and Statistical Analysis

Sociodemographic and anthropometric parameters were presented using descriptive summary statistics, as were data on SAEs and AEs. Data on blood pressure were plotted against time since administration and averaged at each time point of measurement. Similarly, plasma CAR, B-ALA and HIS levels were plotted against time for each subject and averaged. Incremental area under the curve (AUC) was calculated by subtracting the baseline of the AUC calculated using the trapezoid rule. Maximum plasma concentrations (Cmax) were determined as the maximal concentration measured, whereas Tm was the time Cmax was reached. Half-life (T^1/2^) was calculated as 0.693 divided by the elimination constant (Ke), where Ke was calculated by −2.303 × the slope of the individual linear curve of the log10 from Cmax until the concentration at 8 h [21]. Prior to statistical analyses, normality of continuous variables was assessed using the Shapiro–Wilk test. Pearson correlations were performed between PK and anthropometric and dosing parameters. All PK profiles and statistical analyses were performed on IBM SPSS v 27 and GraphPad Prism 10. Values are presented as mean ± SD and significance is assumed at the level < 0.05. Descriptive summary statistics and plots are presented for brain MRI spectroscopic data evaluating CAR concentrations at varying time points.

## 3. Results

Sixteen healthy individuals with no past medical history participated in this study; demographic information is detailed in Table 2. Three quarters were white Caucasian and 25% were Asian, and ranges of age, weight and BMI were wide, indicating an anthropometrically diverse population. Most (68.8%) had no dietary restrictions; however, 12.5% were pescetarian, another 12.5% were vegetarian, and 1 individual (6.2%) was vegan.

### 3.1. Safety and Tolerability

No serious events were reported in any participants, and CAR did not result in any significant haematological or biochemical blood abnormalities at 8 h post-dose, in either the main or long-term dosing sub-study. Table 3 details the frequency of adverse events at the varying doses and the types of adverse events experienced.

No DLT was detected at the 4 g dose, and all participants progressed to the 6 g dose. Only one participant experienced AEs (nausea) at the 6 g dose and did not progress to the 10 g dose, and two further participants did not progress from the 10 g to the 15 g dose owing to AEs (headache, nausea, paraesthesia). However, 10 of the remaining 13 participants taking the 15 g dose experienced AEs and thus met the criteria for DLT, and thus, the DMC and investigators concluded that the study would not progress to the 20 g dose. All AEs reported were rated mild to moderate (Likert 1–3) and only three participants required treatment with simple analgesia for headaches (oral paracetamol or ibuprofen) or an antiemetic (cyclizine) for nausea, all at the 15 g dose. The type of AE experienced was similar across dosing regimens. No AEs were reported within the first hour of ingestion, while 61.5% were experienced between 1 and 4 h after ingestion, and 38.5% between 4 and 12 h after ingestion. Further, the influence of dietary restrictions on experiencing AEs was unclear. The single vegan participant experienced AEs at the 6 g dose, while all three who experienced AEs at the 10 g dose had no dietary restrictions.

Following identification that the 10 g dose was the maximum tolerated dose, four participants underwent the 4-week long-term dosing sub-study at 5 g twice daily and did not report any AEs during the 4-week observation period, nor did they develop any abnormal biochemical or haematological blood abnormalities. It should be noted that two of the participants recruited to the long-term dosing study experienced AEs during the main dose-escalation study at 10 g.

### 3.2. Pharmacokinetics of Carnosine

**Figure 2:**
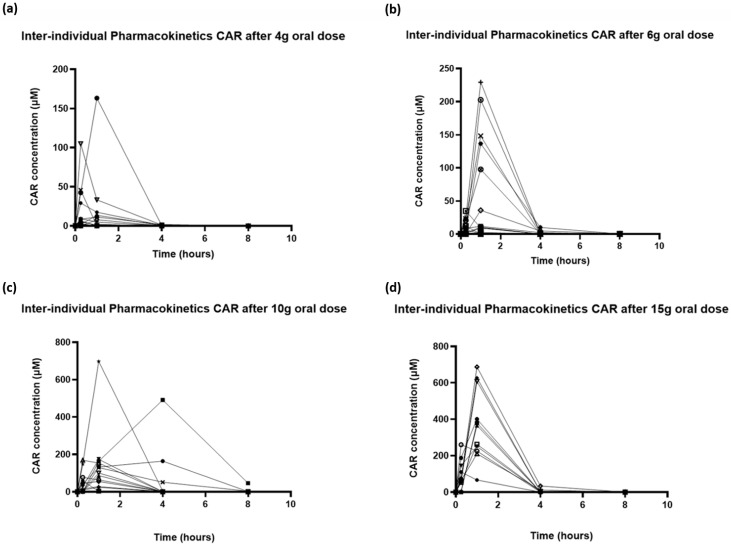
Inter-individual variability in pharmacokinetic profiles of carnosine following oral doses of (**a**) 4 g, (**b**) 6 g, (**c**) 10 g and (**d**) 15 g.

Significant inter-individual variability in plasma concentration profiles was observed for all doses of oral CAR (Figure 2). Increased CAR concentrations in plasma were detectable in 8/16, 12/16, 10/15 and 13/13 subjects at the 4, 6, 10 and 15 g dose levels, respectively. The baseline plasma concentrations of CAR in subjects with measurable baseline CAR ranged between 0.008 and 0.53 μM, and did not vary depending on dietary preferences.

**Figure 3:**
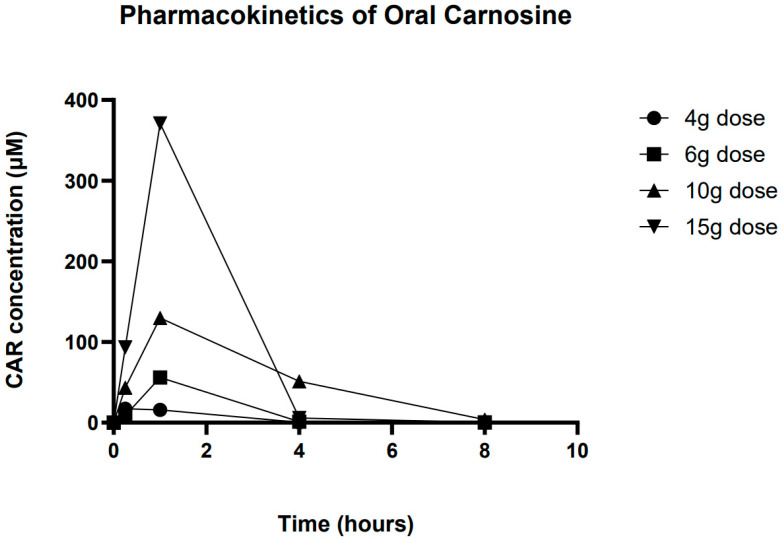
Overall averaged pharmacokinetic profile of carnosine at all 4 doses.

Plotting the mean data for each dose group (baseline subtracted) showed an increase in Cmax and AUC as the dose of CAR was increased (Figure 3). Only at the highest dose were concentrations above baseline values observed at all of the time points. Analysis of the mean concentration time profile showed non-linearity in Cmax and AUC. AUC increased from 39.6 at a 4 g dose to 762.1 at 15 g, while Cmax increased similarly from 17.2 μM to 370.9 μM. Time to Cmax (Tm) was 15 min for 4 g and 1 h for all other doses. Unfortunately, due to the rapid clearance of CAR, for many individuals, detectable levels of CAR were only seen at 15 min and 1 h, meaning that estimation of CAR half-life was not reliable (Table 4).

**Figure 4:**
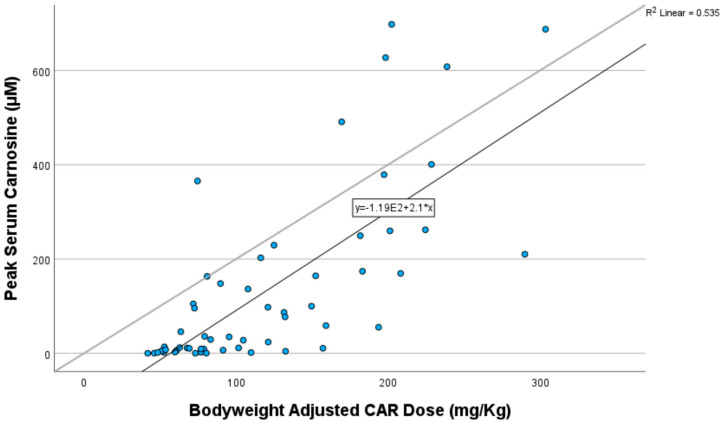
Pearson correlation of peak carnosine concentration with bodyweight-adjusted dose of carnosine taken.

Pearson correlation showed that peak plasma CAR concentrations were strongly correlated with the bodyweight-adjusted dose of oral CAR taken (correlation coefficient 0.732, R^2^ = 0.535, *p* < 0.001) (Figure 4). The PK profile suggests very little CAR is still detectable in the circulation after 4 h of ingestion.

### 3.3. L-Histidine and Beta-Alanine

**Figure 5:**
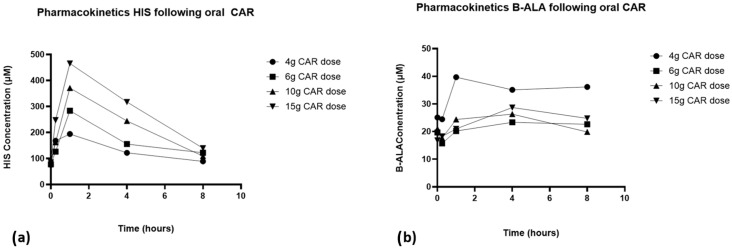
Pharmacokinetic profiles of (**a**) L-histidine and (**b**) beta-alanine at 4 g, 6 g, 10 g and 15 g of carnosine.

Plasma concentrations of breakdown products HIS and B-ALA also varied widely between participants; however, HIS exhibited a PK profile that followed that of CAR and exposure increased with increasing dose, while the PK of B-ALA was more unpredictable (Figure 5). HIS was detectable at baseline in all subjects (levels were 76 +/− 12 mM, 78 +/− 16 mM, 91 +/− 34 mM and 85 +/− 14 mM at the 4, 6, 10 and 15 g CAR doses, respectively), and there was a dose-dependent increase in the Cmax and AUC of histidine with increasing CAR dose, reaching peak concentrations at 1 h (Table 4).

### 3.4. Relationship Between Peak Plasma Concentrations, CAR Dose and Adverse Events

**Figure 6:**
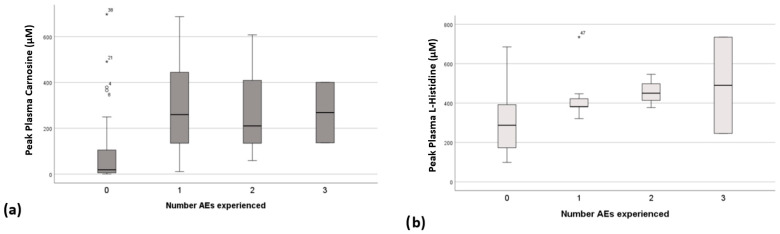
Peak plasma concentrations of (**a**) carnosine and (**b**) L-histidine plotted against number of adverse events experienced with each dose. Outliers figure (**a**) ^o^, and (**b**) *.

Analysis of peak plasma concentrations of CAR and HIS against the occurrence of AEs demonstrates that the number of AEs a participant experiences at a given dose is related to the peak CAR and peak HIS concentrations (Figure 6). Participants were much less likely to develop AEs with peak plasma concentrations of CAR of <150 μM (BWA dose of <100 mg/Kg). There was no clear concentration of HIS below which AEs were unlikely, but a trend towards the experience of multiple AEs with increasing peak plasma concentrations was apparent. No relationship between AEs and B-ALA was observed.

### 3.5. Blood Pressure

**Figure 7:**
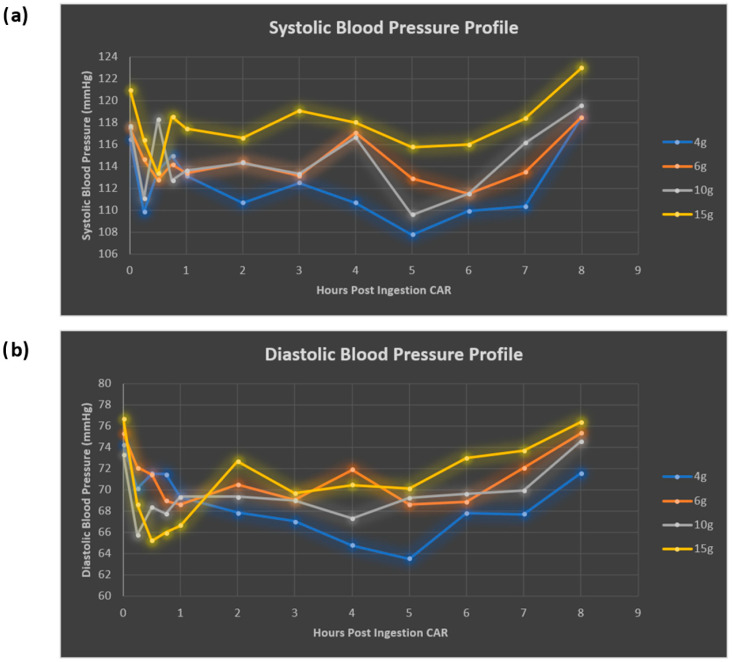
Systolic (**a**) and diastolic (**b**) blood pressure response to oral carnosine ingestion.

Overall, both systolic (SBP) and diastolic (DBP) blood pressure profiles decreased following CAR ingestion, with the greatest reductions seen following the 4 g dose (8.7 mmHg drop SBP and 10.7 mmHg drop DBP from baseline). Early reductions and fluctuations were observed in both SBP and DBP within the first hour, and BP nadirs occurred between 5 and 6 h (Figure 7, Table 5). Both SBP and DBP returned to baseline by 8 h. Interestingly BP reduction did not correlate with the occurrence of headache. No changes were noted with HR or RR.

### 3.6. Brain Carnosine Concentrations

**Figure 8:**
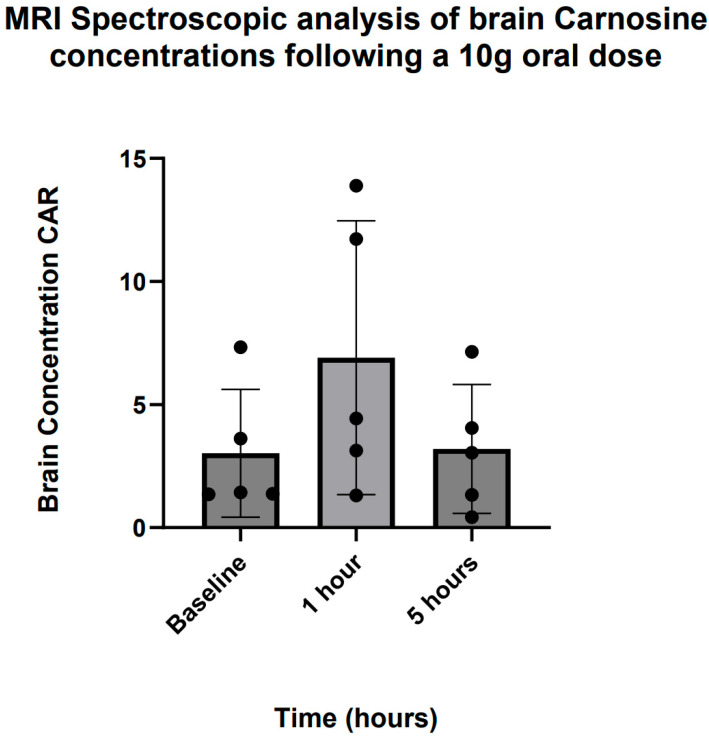
MRI spectroscopic analysis of brain carnosine concentrations following a 10 g oral dose.

Proton MRI spectroscopic analysis revealed increased detection of brain CAR levels 1 h following ingestion of a 10 g oral dose in five participants (Figure 8), with a return to baseline by 5 h. Due to the small sample size, statistical inference was not possible, but qualitatively, the dynamic pattern of CAR in the brain was highly consistent with that found in plasma.

## 4. Discussion

This dose-escalation study is the first of its kind to identify the maximum tolerated dose of oral CAR in a healthy human population. Furthermore, the use of novel biochemical analytical methods has enabled an accurate understanding of the pharmacokinetic profiles of CAR and its breakdown products following oral fixed-dose strategies. These results suggest that oral CAR up to a dose of 10 g is safe and well tolerated. Beyond this, side effects occur at an unacceptable rate.

### 4.1. Safety and Tolerability

There is surprisingly little literature relating to the side effect profile of orally administered CAR supplementation. Prior to this study, analysis of blood CAR responses to oral CAR involved much smaller doses, either given as food products, e.g., chicken broth (~450 mg CAR) [21] or ground beef (~250 mg) [16], or supplements up to 4 g [17,24,25]. None of the above studies report side effects amongst participants, which is in keeping with our findings for the 4 g dose. Interestingly, Everaert et al. [17] were only able to detect increased plasma CAR concentrations (>10 μM from baseline) in 8 of the 25 healthy participants following ingestion of a 4 g dose of oral CAR (mean 60 mg/Kg BWA dose), deemed ‘responders’, while 8 of our 16 participants (50%) met this criterion. They also reported higher concentrations of B-ALA at 1 h post-dose (200–300 μM) compared to our findings at 1 h (30–40 μM). While this may be due to differences in gut absorption or tissue distribution and metabolism, it is likely to be due to the effect of pre-treating our cooled EDTA blood collection tubes with carnostatine, reducing the effect of post-collection carnosinase activity on degrading CAR to its metabolites HIS and B-ALA.

At an oral dose of 10 g, 3 of 15 (20%) participants experienced adverse events, which is a comparable rate to adverse events reported in placebo arms of interventional anti-depressant trials, for example [26]. All AEs reported at 10 g (mean 146.7 mg/Kg BWA) were mild, transitory and did not require specific treatment. In contrast, 77% of participants at the 15 g dose (220.2 mg/Kg BWA) experienced AEs. When analysing the peak plasma concentrations and risk of AEs with each dose of CAR given, the risk of developing AEs appeared much greater with peak plasma concentrations of >150 μM, which equated to an approximate BWA dose of 125 mg/Kg from our correlation. Of the AEs reported in this study, headache, nausea and pruritis have been reported in previous CAR studies [27,28]. While the majority of AEs in this study occurred within the first 4 h, over a third occurred between 4 and 12 h post-dose. As very little CAR is left in the circulation at this point, it suggests that mechanistic pathways leading to AEs may have been triggered early by CAR and take time to develop, or that these later AEs are related to its breakdown products HIS or B-ALA. In particular, HIS levels remained elevated up to 8 hrs following the 10 g and 15 g doses, and HIS has been reported to cause headache and nausea [29], while the literature suggests B-ALA supplementation can lead to paraesthesia and flushing [30,31]. Thus, identifying precisely whether it is CAR or its metabolites that are responsible for AE occurrence may be difficult; however, the transitory nature of the AEs is encouraging. Further, the lack of AE occurrence in the four participants undergoing long-term dosing supports the safety and tolerability of the 5 g twice-daily dose in longer-term use. These data, coupled with PK profiles, are valuable when considering the potential of CAR for neuroprotection and neurodegeneration. For instance, the neuroprotective role of CAR in preclinical models of ischaemic stroke has been investigated at doses of between 500 and 2000 mg/Kg administered intravenously [8]. Thus, it is clear that the maximum tolerated oral doses in this study (10 g or 146.7 mg/Kg) will fall short of achieving relatively similar circulating and tissue concentrations seen in these animal models; thus, investigating intravenous routes of CAR administration may be necessary in human neuroprotection studies. However, a recent review of clinical randomised controlled trials of CAR in the management of neurodegenerative and psychiatric conditions such as Alzheimer’s d, Parkinson’s disease, autism spectrum disorder, schizophrenia and attention-deficit hyperactivity disorder found oral CAR dosing regimens that ranged from 200 mg to 2 g daily [32]. Thus, long-term dosing strategies commonly used in such clinical trials may benefit from safe and tolerable dose increments if clinical efficacy is lacking.

### 4.2. Blood Pressure Effects

The data in this study suggests that oral CAR dosing results in a two-phase reduction in both systolic and diastolic BP, an initial rapid reduction in BP within the 1st hour, and a second gradual reduction over 1–5 h followed by a recovery to baseline by 8 h. These reductions did not appear dose-dependent and were actually most profound at the 4 g dose, although it is possible that the occurrence of AEs such as headache and nausea at higher doses activated sympathetic pathways that led to lower BP reductions. While a dose-dependent vasodilatory effect of CAR has previously been demonstrated in rat aortic smooth muscle [33], it is much more likely that the antihypertensive action of CAR is mediated via the decarboxylation of HIS to histamine that acts on the H1 receptor pathways that lead to vasodilatation [34]. If such vasodilatory effects were to occur within the cerebral vasculature, these effects may be of great interest in neurodegenerative disorders such as Alzheimer’s disease that are associated with reduced cerebral perfusion states [35]. Hisatsune et al. demonstrated modest preservation of cerebral blood flow in the posterior cingulate gyrus in elderly individuals at risk of dementia taking CAR supplementation (1 g daily for 3 months) compared to placebo, which was associated with preservation of delayed recall [36]. One may postulate that the cerebral vasodilatory effect of oral CAR may be responsible for the relatively common occurrence of headache at higher doses. If the changes in SBP and DBP we observed were however real, it may mean that caution is exercised in using high doses of CAR in the target elderly neurodegenerative population, where higher rates of orthostatic hypotension and blood pressure variability may increase the risk of presyncope and falls [37]. However, data on the haemodynamic effect of CAR on systemic BP is conflicting, as a recent randomised controlled trial of oral CAR (1 g twice daily, 14 weeks) did not demonstrate any long-term effect on either systolic or diastolic BP in people at risk of or who had type 2 diabetes [38].

### 4.3. Brain Penetration

Findings from this study suggest that rapid accumulation of brain CAR can be seen after oral ingestion of a 10 g dose, in a pattern not dissimilar to plasma. MRI spectroscopy detected baseline CAR levels in the brain. While most of the CAR in the brain is assumed to come from de novo synthesis (both HIS and B-ALA can cross the blood–brain barrier, BBB) in glial cells [32], dietary CAR can also cross the BBB, as evidenced by our findings. Brain penetration studies of CAR using MRI spectroscopy are scarce, with most focus to date in skeletal muscle [39]. Solis et al. found that 4 weeks of B-ALA supplementation at 6.4 g daily did not lead to any detectable rise in brain CAR levels from baseline using 3T brain MRI spectroscopy in seven trained cyclists [40]; however, this is unsurprising given that the rise in brain CAR concentration reverted to baseline levels within 5 h in our study. If CAR is having an effect on brain metabolic or immune function, it is triggering pathway cascades before being metabolised itself by elevated carnosinase activity.

### 4.4. Limitations

Our study was prone to several possible limitations. Firstly, this was a small study, with even fewer participants in the brain concentration and long-term dosing sub-studies, and thus prone to small-study effects [41], especially given the significant inter-individual variability in PK response. Second, CAR is broken down rapidly following blood draw, and thus, we may have underestimated plasma CAR concentrations consequently; however, our proficient sample acquisition procedures and utilisation of carnostatine-pre-treated EDTA collection tubes helped mitigate this as much as possible. Third, while we concentrated on the posterior cingulate gyrus as a region of interest for brain MRI spectroscopy, it is unclear if this is the most sensitive area of the brain to demonstrate change in CAR concentration. Furthermore, MRI spectroscopy image processing is prone to variation, although we aimed to adhere to published standardised protocols. Also of importance is that, while we may use techniques such as MRI spectroscopy to detect changes to brain CAR levels, we are unclear yet if these changes are physiologically meaningful. Fourth, we accept that variations in the PK profile parameters calculated (e.g., T^1/2^ CAR) may stem from the limited number of data points available to create more accurate representations of CAR PK profiles; however, they provide useful estimations in this population. Fifth, while we demonstrated a relatively linear relationship between CAR dosing and plasma concentrations from correlation analysis, we cannot exclude a two-stage relationship where plasma concentrations only rise once enzymatic processes, such as carnosinase-dependent CAR breakdown, become saturated. Further analysis of a larger sample size including analysis of endogenous carnosinase activity would aid understanding here. Sixth, these data are indicative of PK profiles in fasted states. Absorption and thus time to Cmax may be delayed in non-fasted states due to digestive processes and competition for absorptive transportation carriers with other enteral nutrients. Seventh, our carnostatine stability experiments involved incubation of the carnostatine/EDTA with whole blood (rather than plasma) for 45 min at 37 degrees Celsius; however, we did not allow such incubation of samples taken from participants during the study. This was because we wanted to reduce the breakdown of CAR by endogenous carnosinases as much as possible while the plasma was being prepared, but we acknowledge that the stabilising effect of carnostatine in our stability report may not reflect precise events during the trial sampling and processing. Finally, while our population is much younger than the average age of populations who have neurovascular or neurodegenerative conditions, a recent systematic review demonstrated that oral CAR at lower doses was tolerated in older individuals with delayed recall [25], suggesting that the use of these higher doses could be explored.

## 5. Conclusions

Carnosine at an oral dose of up to 10 g is safe and well tolerated, in a single dose and in divided doses as a long-term dosing strategy, in the majority of individuals. At doses of 15 g, the frequency of adverse events becomes unacceptably high, with 77% of participants experiencing side effects, most commonly headache (43.5%), nausea (21.7%) and paraesthesia (21.7%). Single doses result in elevations in plasma and brain CAR concentrations, although rapid metabolism means baseline levels are re-established within 4–5 h, while the breakdown product HIS takes longer to clear. In addition, oral CAR administration may also have acute BP-lowering properties via its direct effect on smooth muscle or via histamine receptor pathways. Overall, these data provide the first insights into the maximum tolerated doses of oral CAR and its side effect profiles, and may be used to design optimal CAR dosing strategies for investigation in future clinical trials.

## Figures and Tables

**Table 1:** Participant inclusion and exclusion criteria.

Inclusions	Exclusions
Age 18 years or over	On regular medications (except contraception)
On established contraception if female and of childbearing age	History of medical or mental health illnesses
	Contraindications to MRI scanning
	Pregnant or breastfeeding
	History of smoking or substance misuse
	Known allergy to CAR, B-ALA or HIS
	Known allergy to meat or fish products
	BMI of >30
	Resting BP of <100/70 mmHg

MRI = magnetic resonance imaging; CAR = carnosine; B-ALA = beta-alanine; HIS = L-histidine; BMI = body mass index (Kg/m^2^).

**Table 2:** Study participant demographic and anthropometric characteristics.

	All Participants (*n* = 16) (Mean ± SD)	Range
**Age (Yrs)**	29.4 (10)	22–56
**Sex (%)**		
Male	37.5	
Female	62.5	
**Weight (Kg)**	70.7 ± 13.8	49–95
**BMI (Kg/m^2^)**	24.4 ± 3.5	19.7–29.7
**Ethnicity (%)**		
White Caucasian	75	
Asian	25	
**Diet (%)**		
Meat eater	68.8	
Pescetarian	12.5	
Vegetarian	12.5	
Vegan	6.2	
**Baseline CrCl (mL/min)**	96.2 ± 11.4	72.1–105.6
**Bodyweight-adjusted (BWA) dosing (mg/Kg)**		
4 g	58.7 ± 12.1	41.9–81.0
6 g	88.1 ± 18.2	62.9–121.0
10 g	146.7 ± 30.6	104.8–202.4
15 g	220.2 ± 45.6	157.2–303.6

BMI = body mass index; CrCl = creatinine clearance.

**Table 3:** Summary of adverse event profiles experienced by participants at all doses.

Adverse Events Experienced at Each Dose Escalation		
**Dose**	**N**	**%**
4 g	0/16	0
6 g	1/16	6.3
10 g	3/15	20
15 g	10/13	77
Long-term dosing 5 g bd	0/4	0
**Adverse events (AEs) and their prevalence**		**% AEs**
Headache		43.5
Nausea/vomiting		21.7
Paraesthesia		21.7
Hypotension		4.3
Flushing		4.3

N = number of participants; AEs = adverse events.

**Table 4:** Summary pharmacokinetics for area under the curve (AUC), peak plasma concentrations (Cmax), time to peak concentrations (Tm) and half-life (T ^1/2^) for differing carnosine doses. *AUC units* μM × hours (μM * h).

Carnosine Pharmacokinetic Parameters				
Carnosine Dose	AUC (μM * h) (±SD)	Cmax (μM) (±SD)	Tm (min)	T ^1/2^ (min)
4 g	39.6 ± 82.6	17.2 ± 45.4	15	NA
6 g	113.0 ± 153.7	56.0 ± 78.1	60	NA
10 g	450.6 ± 610.5	129.8 ± 195.5	60	NA
15 g	762.1 ± 934.6	370.9 ± 428.2	60	NA
**L-Histidine pharmacokinetic parameters**				
Carnosine Dose	AUC (μM * h) (±SD)	Cmax (μM) (±SD)	Tm (min)	T ^1/2^ (min) (±SD)
4 g	456.7 ± 563.8	194 ± 243.2	60	130.8 ± 152.4
6 g	786.9 ± 921.9	283.6 ± 416.4	60	85.2 ± 98.3
10 g	1256.0 ± 1482.5	371.0 ± 487.3	60	192.0 ± 213.7
15 g	1791.0 ± 1993.5	465.3 ± 572.9	60	246.0 ± 298.6

NA = not applicable.

**Table 5:** Blood pressure responses following oral carnosine ingestion.

Systolic Blood Pressure Drop from Baseline at Nadir		
**Carnosine Dose**	**Blood Pressure Drop (mmHg)**	**Blood Pressure Drop (%)**
4 g	8.7	7.5
6 g	6	5.1
10 g	8.2	7
15 g	5.2	4.3
**Diastolic Blood Pressure Drop from Baseline at Nadir**		
**Carnosine Dose**	**Blood Pressure Drop (mmHg)**	**Blood Pressure Drop (%)**
4 g	10.7	14.4
6 g	6	8
10 g	6.1	8.2
15 g	7	9.1

mmHg = millimetres of mercury.

## Data Availability

The datasets collected and analysed during the current study can be made available on reasonable request from the corresponding author. The datasets are not publicly available due to being part of an ongoing study.

## Supplementary Materials

The following supporting information can be downloaded at https://www.mdpi.com/article/10.3390/nu17132130/s1. A carnosine stability report. References [42,43] are cited in Supplementary Materials.
