# Supplementary material for: Dietary Carnosine Supplementation in Healthy Human Volunteers: A Safety, Tolerability, Plasma and Brain Concentration Study
Source: Nutrients. 2025 Jun 27;17(13):2130. doi: 10.3390/nu17132130 (PMC12252343; doi:10.3390/nu17132130)
Supplement: Supplementary file 1 [file nutrients-17-02130-s001.zip › nutrients-3678679-supplementary.pdf]

Supplementary data.

Detection of Carnosine, Histidine and Alanine in Human Plasma Samples Pre-treated with Carnostatine

29th April 2025

By Amy Grayson, on behalf of the Sheffield University

Prepared for: Prof. Arshad Majid, Sheffield Institute for Translational Neuroscience (SITraN), University of Sheffield

Aims

The aim of this study was to measure the concentration of L-carnosine, L-alanine and L-histidine in serum samples pre-treated with carnostatine, a carnosinase inhibitor.

Materials and Methods

Blood samples (4ml from the same individual) were taken at room temperature approximately 1 hour after lunch, from the ante-cubital fossa, into ice cooled EDTA tubes pretreated with 0.905mg carnosine (to give a concentration of 1000 uM) and differing concentrations of carnostatine (see table 1), a carnosinase inhibitor, aimed at reducing breakdown of carnosine during sample processing. Seven different concentrations of carnostatine were investigated, 0, 0.1, 1.0, 10.0, 20.0, 50.0 and 100.0 uM respectively. While a known quantity of carnosine (CAR) was added to each sample, the natural level of CAR in the blood 1 hour following lunch was unknown, but would mean that the real concentration of CAR in the samples would be higher than the added CAR dose. Thus recovery of CAR at different carnostatine doses could theoretically surpass 100% of the added CAR dose. This was acceptable as we were interested in the optimal level of CAR recovery at differing carnostatine doses. These samples were transported to the laboratory immediately and spun in a refrigerated (4°C) centrifuge (Eppendorf Centrifuge 5804R) at 13,000 rpm for 10 minutes to prepare plasma for storing at -80 degrees.

**Carnostatine (µM)**	**Added Carnosine Dose Concentration (uM)**
0.0	1000.0
0.1	1000.0
1.0	1000.0
10.0	1000.0
20.0	1000.0
50.0	1000.0
100.0	1000.0

Table 1. Representation of the concentrations of carnostatine and carnosine in the blood samples after addition of 4ml whole blood. The seven different concentration conditions were used to investigate the optimal concentration of carnostatine needed for optimal carnosine recovery.

Frozen plasma samples were later thawed at room temperature, vortex mixed and centrifuged at 13,000 rpm for 20 minutes at 4°C. 25 µL of supernatant was removed and 25 µL of each heavy-labelled internal standard (25 µL of 500 µM DL-histidine-d3, 25 µL of 500 µM L-carnosine-d4, 25 µL of 500 µM β-alanine-$^{13}C_3{}^{15}N$, all prepared separately in water with 0.1% formic acid) was added, to give a final volume of 100 µL. 150 µL of acetonitrile with 0.1% formic acid solution was added to each sample. Samples were then vortex mixed and centrifuged at 13,000 rpm for 20 minutes at 4°C. 10 µL of supernatant was mixed with 390 µL of 60% acetonitrile containing 0.1% formic acid and placed in LC-MS vials for analysis. 10 µL of each sample was injected and analysed in a multiple reaction monitoring (MRM) assay using liquid chromatography (LC)-coupled tandem mass spectrometry (MS/MS) with an electrospray ionisation (ESI) source (LC-ESI-MRM-MS/MS).

LC-ESI-MRM-MS/MS

Normal phase liquid chromatography was performed using an Agilent Infinity II 1290 high performance liquid chromatography (HPLC) system. Solvent A (100% HPLC-grade water with 0.1% formic acid) and solvent B (100% acetonitrile with 0.1% formic acid) were used along with an XBridge BEH Amide 130Å, 5 µM, 4.6 x 150 mm column (Waters Ltd, UK), with the column temperature set to 30°C. Peptides were eluted at 0.4 mL/min from 90%-20% B over 16 minutes, 20% to 5% B over 0.3 minutes, held at 5% B for 2 minutes, 5% B to 90% over 0.7 minutes then held at 90% B for 10 minutes. An Agilent Ultivo triple quadrupole mass spectrometer was used with Agilent Jet Stream Electrospray Ionisation (AJS-ESI) source. Electrospray ionization was performed in positive polarity mode, with source parameters as follows: sheath gas temperature = 250°C; sheath gas flow rate = 11 L/min; desolvation gas temperature = 300°C; desolvation gas flow rate = 7 L/min; nebuliser gas pressure = 15 psi; capillary voltage = 4000 V; nozzle voltage = 1500 V. The mass spectrometer was operated in a dynamic multiple reaction monitoring (dMRM) mode. Table 2 contains details of the transitions used.

Target	Precursor m/z	Product m/z	Retention Time (mins)	Retention Window (mins)
L-carnosine	227.1	110.1	15	4
L-carnosine-d4	231.1	156.1	15	4
L-histidine	156.1	110.1	14.8	4
DL-histidine-d3	159.2	113.2	14.8	4
L-alanine	90.1	44.1	12	4
β-alanine-$^{13}C_3{}^{15}N$	94.1	32.1	12	4

Table 2. Details of multiple reaction monitoring transitions analysed for each target compound.

Raw mass spectrometry data was collected using MassHunter Workstation LC/MS Data Acquisition version 1.2 software and analysed using MassHunter Workstation Qualitative Analysis version 10.0 software (Agilent Technologies Inc, California, USA).

Method Optimisation

Fragmentor voltage and collision energy were optimised for each MRM target to increase assay sensitivity. 10 µL of each standard was injected in triplicate at a range of fragmentor voltages in 10 V steps, and at the default voltage of 135 V. Once the fragmentor voltage was optimised for each compound, the method was adjusted accordingly, and the collision energy was optimised for each transition. 10 µL of each standard was injected in triplicate at a range of collision energies from 0 to 40 V.

Standard Curves and Quality Control

Serial dilutions of each standard (L-carnosine, L-carnosine-d4, L-histidine, DL-histidine-d3, L-alanine, and β-alanine-$^{13}C_3{}^{15}N$) were prepared to create a series of 11 standards ranging from 0.025 to 5 µM. Standards were injected in a randomised order at the beginning and end of each experimental run, and after analysis of each sample set. 10 µL of blank sample containing 60% acetonitrile with 0.1% formic acid was injected at the beginning and end of each experimental run, before and after each set of standards, and after every 10 sample injections.

Results:

Reviewer comment: The method validation is not convincing enough. Such a bioanalytical method should be described in more detail, especially if it has not been published yet. Both the FDA and EMA provide detailed guidelines on bioanalytical method validation - accuracy, precision, linearity, and stability. If the authors have described such details in another paper - please cite. If not, please consider adding such information as a supplementary file.

Response:

Data Validation:

Accuracy and Precision

Under optimised LC-MS/MS conditions, plasma samples showed no significant interfering peaks at the retention times for each target compound (Table 1 and Figure 1).

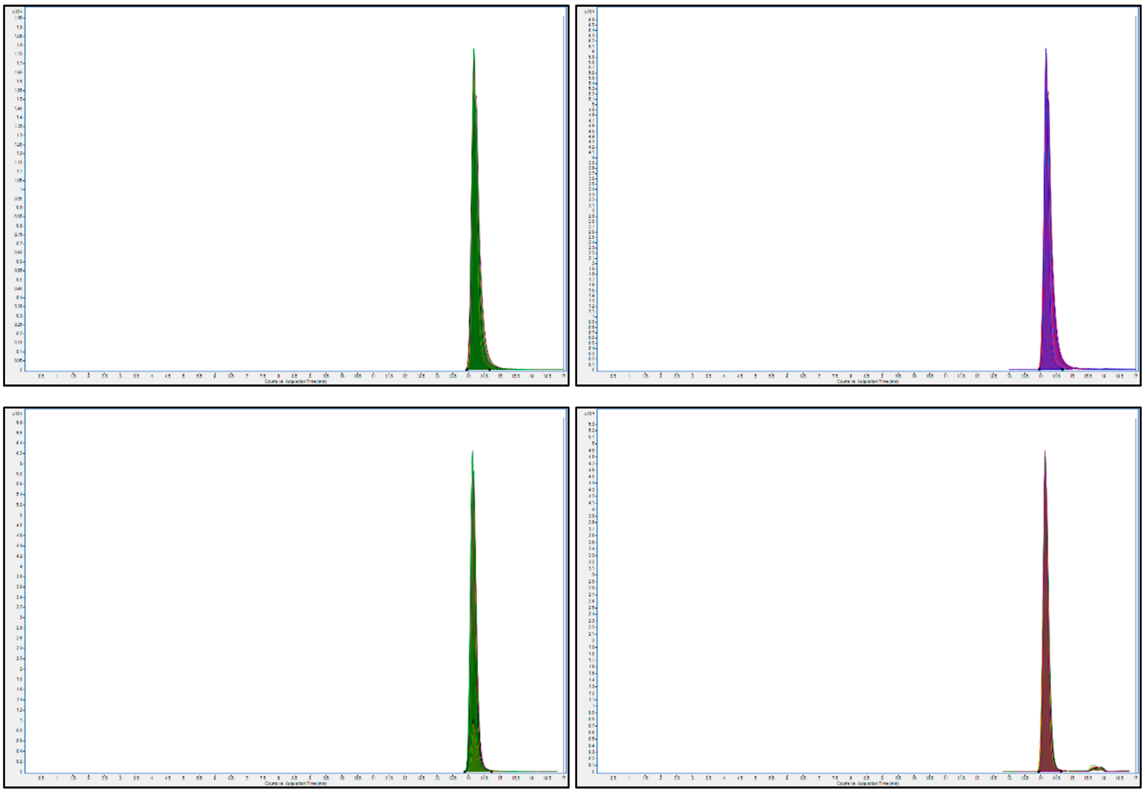

Figure 1. Chromatograms of a) L-carnosine, b) L-carnosine-d_4, c) L-histidine, d) DL-histidine-d_3 in 11 standards and 7 samples in triplicate analysis (n=3), showing peak area and retention time.

The results in Figure 1 show that L-carnosine-d4 and DL-histidine-d3 heavy labelled standards eluted at the same time within the standards and the plasma samples. In addition, the light-labelled compounds (L-carnosine and L-histidine) within the standards and the native compounds found within

the plasma samples also gave the same retention time. This suggests that the LC method has high reproducibility, robustness, selectivity and specificity.

Additionally, the chromatograms for the matched heavy- and light-labelled standards were overlapped to investigate the accuracy of the LC method. Figure 2 below shows the overlapping chromatography of matched heavy and light labelled compounds analysed by LC-MRM-MS.

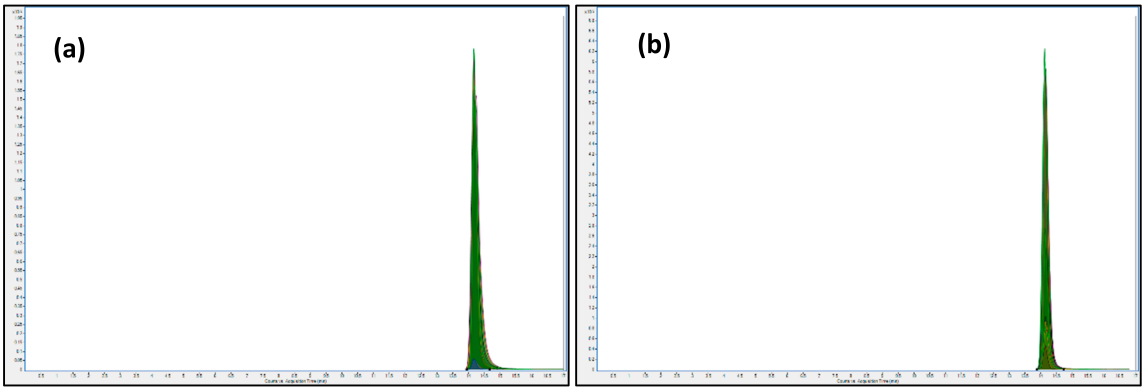

Figure 2. Overlapping chromatography of a) L-carnosine and L-carnosine-d4 and b) L-histidine and DL-histidine-d3 in 11 standards and 7 samples in triplicate analysis (n=3), showing peak area and retention time.

The results in Figure 2 show that the chromatographic peaks for (a) L-carnosine and L-carnosine-d4 and (b) L-alanine and DL-alanine-d3 have comparable retention times as would be expected for a heavy labelled standard of the same molecule.

The variance in the peak area measurement (CV%) was calculated by measurement of the peak area for each target compound in seven plasma samples, analysed in triplicate. The peak area precision of these samples ranged from 2.88 – 6.41% for L-carnosine and 0.50 – 2.90% for L-histidine. All CV values were ≤ 15%, which is within acceptable ranges according to US FDA and EMA guidelines (European Medicines Agency, 2022; Food and Drug Administration, 2022).

To investigate the precession of the LC-MRM-MS/MS method, ten repeated injections of the same test sample was analysed in succession with standard curves being generated either side of the acquisitions. The average concentration of each target analyte was reported with standard error of the mean (SEM) (Figure 3). The regression line of the mean values of the two standard curves run

either side of the ten successive injections was used to calculate the concentration of each analyte in the original sample.

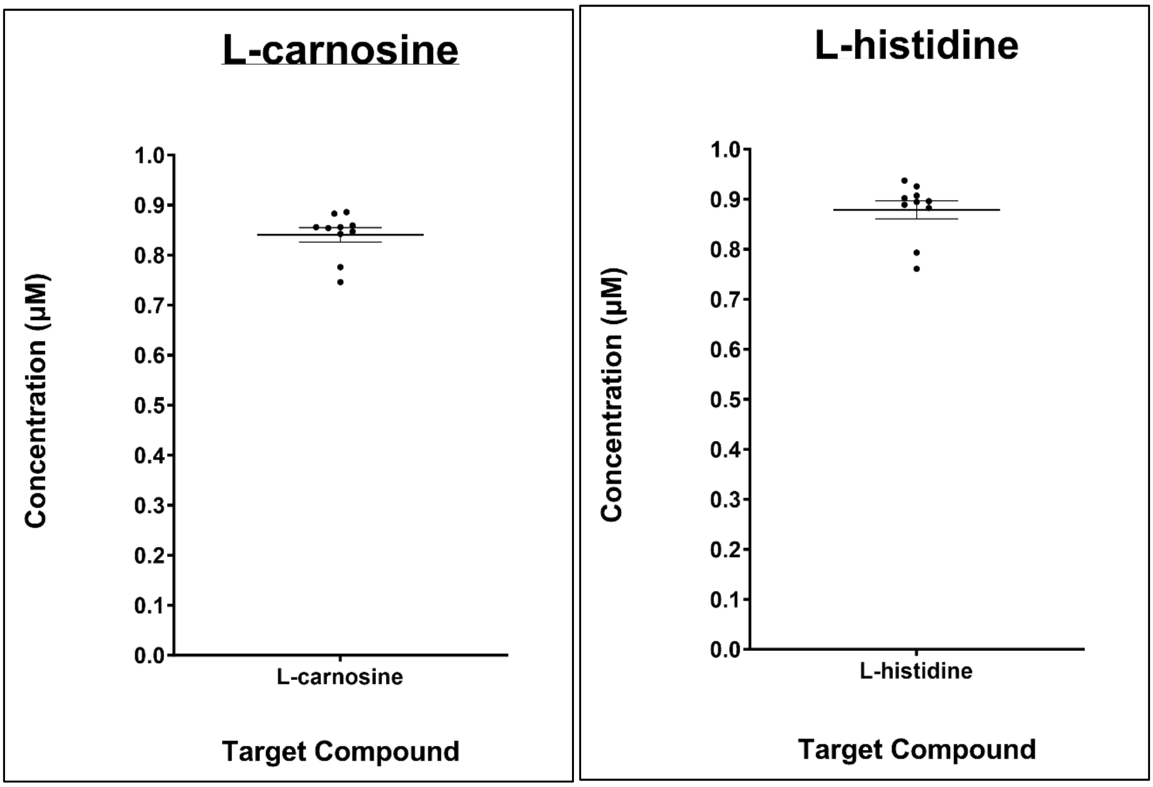

Figure 3. Dot plot of the average (mean) calculated concentration of a) L-carnosine, b) L-histidine and c) L-alanine in 10 repeated injections. Error bars indicate standard error of the mean.

The precision of the method for determining L-carnosine and L-histidine concentrations were within the acceptable ranges according to the United States Food and Drug Administration (US-FDA) and European Medicines Agency (EMA) guidelines ((European Medicines Agency, 2022; Food and Drug Administration, 2022), as the CV% for L-carnosine was reported to be 5.32% while the CV% for L-histidine was 6.44%, indicating the suitability and reproducibility of the analytical method at the specified concentration range.

Linearity and Reproducibility

For linearity evaluation, the calibration curve containing a mixture of all six light and heavy-labelled target compounds was analysed in triplicate over 35 hours over the range of 0.025 – 5 µM. 10 calibration curve points were analysed using the developed method. The calibration curves were drawn by plotting the peak area ratio of analyte to IS versus the nominal concentration of each analyte.

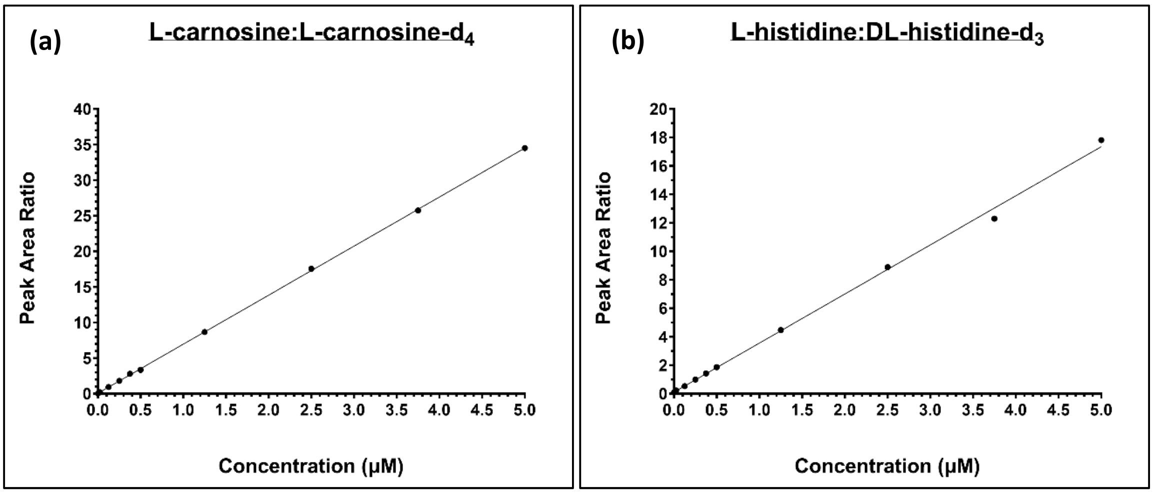

Figure 4. (a) L-carnosine:L-carnosine-d4, y= 6.890x + 0.06245, R^2 = 0.9999. (b) L-histidine:DL-histidine-d3, y=3.448x + 0.01093, R^2 = 0.9976

To test the linearity and reproducibility of the assay, serial dilutions of IS and target compound standards were analysed in triplicate over a total of 35 hours. Four standard curves were produced from 11 serially diluted samples containing the target compounds L-carnosine, L-histidine and L-alanine and their heavy-labelled IS counterparts L-carnosine-d4, DL-histidine-d3 and β-alanine-$^{13}C_3{}^{15}N$ respectively.

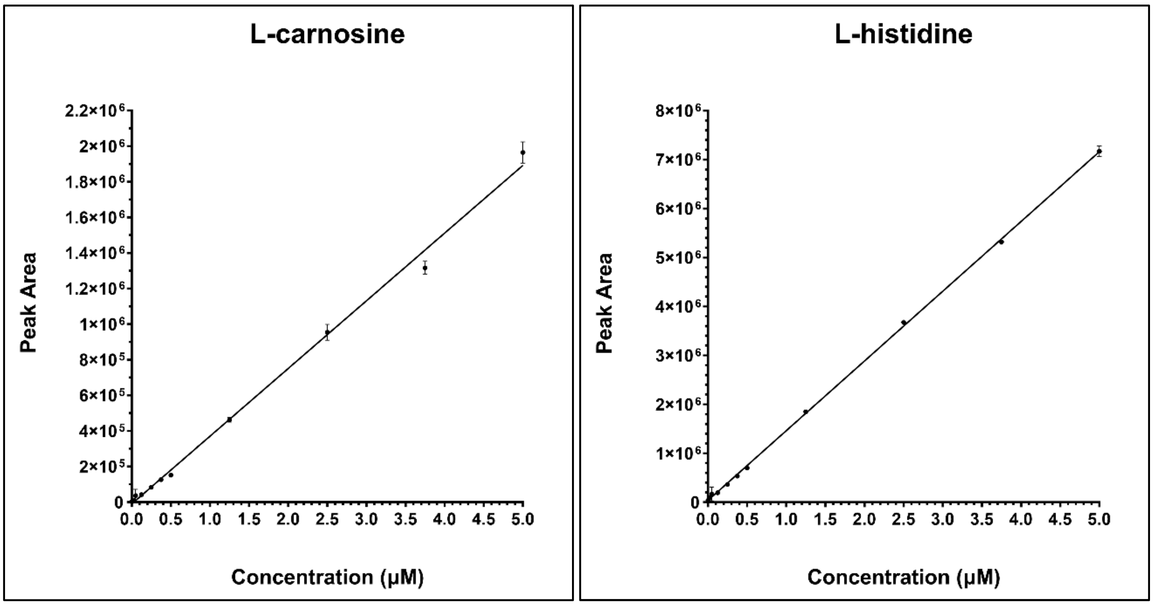

Figure 5. Calibration curves produced by analysis of 11 serially diluted samples of target compounds (standards) spiked with internal standard (IS) and quantified by the LC-MRM-MS/MS assay (n=4). L-carnosine linear regression: y=380410x – 10157, R^2 = 0.9945. L-histidine linear regression: y=1424922x + 36462, R^2 = 0.9997.

Figure 5 above shows the repeated analysis of four standard curves of different concentrations of each compound and suggest the LC-MRM-MS assay has high reproducibility and linearity over the range of 0.025 – 5 µM and over 35 hours.

Figure 6 below shows the peak area when analysing repeated injections of the same sample over time to measure instrument stability.

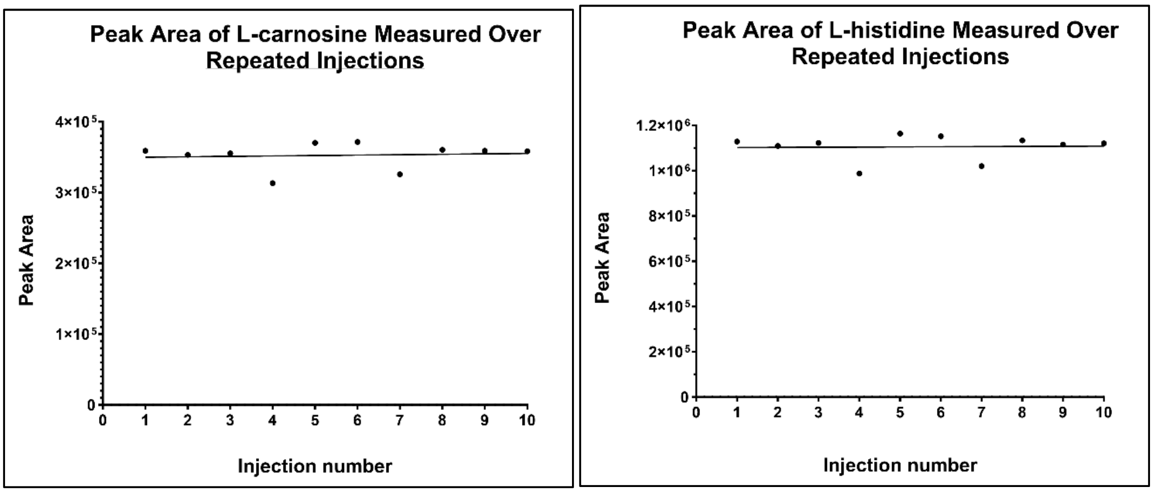

Figure 6. Instrument stability of measuring a single calibration standard in ten replicate injections. A) L-carnosine, y=586.3x + 343462, R^2 = 0.009110. B) L-histidine, y=715.06x + 1093967, R^2 = 0.001465.

In terms of CAR recovery from the seven different carnostatine concentration samples, recovery peaked at 20.0 and 50.0 uM at 105.0 and 104.0% respectively (table 3).

Carnostatine (µM)	Carnosine Dose Concentration (uM)	Recovered Carnosine Concentration (uM)	% Recovered
0.0	1000.0	197.7	20.0
0.1	1000.0	77.6	8.0
1.0	1000.0	677.1	68.0
10.0	1000.0	935.3	94.0
20.0	1000.0	1045.4	105.0
50.0	1000.0	1043.2	104.0
100.0	1000.0	990.1	99.0

Table 3. Carnostatine addition and its effects on recovery of Carnosine.

References:

European Medicines Agency. (2022). *ICH guideline M10 on bioanalytical method validation and study sample analysis*. www.ema.europa.eu/contact

Food and Drug Administration. (2022). *M10 BIOANALYTICAL METHOD VALIDATION AND STUDY SAMPLE ANALYSIS Guidance for Industry*. https://www.fda.gov/regulatory-information/search-fda-guidance-documents/m10-bioanalytical-method-validation-and-study-sample-analysis

Supplementary data - Details of multiple reaction monitoring transitions analysed for each target compound.

**Analyte**	**Transitions**	**Retention Time (mins)**	**Fragmentor Voltage (V)**	**CE (V)**	**Retention Window (mins)**
L-carnosine	227.2→110.1	15.1	135	20	4.0
L-carnosine-d4	231.1→156.1	15.1	135	20	4.0
L-histidine	156.2→110.1	14.8	135	20	4.0
DL-histidine-d3	159.2→113.2	14.8	135	20	4.0
L-alanine	90.1→33.1	12.0	135	20	4.0
β-alanine $3C^{13}$ N^{15}	94.1→32.1	12.0	135	20	4.0

CE = collision energy
